# Crystallin gene mutations in Indian families with inherited pediatric cataract

**Published:** 2008-06-16

**Authors:** Ramachandran Ramya Devi, Wenliang Yao, Perumalsamy Vijayalakshmi, Yuri V. Sergeev, Periasamy Sundaresan, J. Fielding Hejtmancik

**Affiliations:** 1 Department of Genetics, Dr. G. Venketaswamy Eye Research Institute, Aravind Medical Research Foundation, Aravind Eye Hospital, Madurai, India; 2 Ophthalmic Genetics and Visual Function Branch, National Eye Institute, National Institute of Health, Bethesda, MD; 3 Pediatric Clinic of Ophthalmology, Aravind Eye Hospital, Madurai, India

## Abstract

**Purpose:**

Pediatric cataract is the most common form of treatable childhood blindness and is both clinically and genetically heterogeneous. Autosomal dominant and recessive forms of cataract have been reported to be caused by mutations in 22 different genes so far. Of the cataract mutations reported to date, about half the mutations occur in crystallins, a quarter of the mutations in connexins, and the remainder is evenly divided between intrinsic membrane proteins, intermediate filament proteins, and transcription factors. This study is aimed at identification of the spectrum and frequency of crystallin gene mutations in cataractous patients in an Indian population.

**Methods:**

Genetic analysis was extended to screen the entire coding region of the *CRYAA*, *CRYAB*, *CRYBA1*, *CRYBA4*, *CRYBB1*, *CRYBB2*, *CRYBB3*, *CRYGC*, *CRYGD*, and *CRYGS* genes using single stranded conformational polymorphism (SSCP) analysis as a screening technique followed by direct sequencing of all subjects that displayed an electrophoretic shift.

**Results:**

This report describes the first simultaneous mutation analysis of 10 crystallin genes in the same population, represented by 60 south Indian families. The analysis allowed the identification of causative mutations in 10 of the families (three novel and six reported). This includes six missense mutations (CRYAA-R12C, R21W, R54C, CRYAB- A171T, CRYGC-R168W, CRYGS- S39C), two nonsense mutations (CRYBB2- Q155X, CRYGD- R140X), and one splice mutation, which was identified in two families (CRYBA1-IVS3+1G>A).

**Conclusions:**

Crystallin mutations are responsible for 16.6% of the inherited pediatric cataract in this population. As causative mutations have not been found in many of the families analyzed, this study suggests the presence of further novel genes or sequence elements involved in the pathogenesis of cataract in these families.

## Introduction

Pediatric cataracts are common and represent one of the most treatable causes of lifelong visual impairment. It is estimated that globally, 20 million children under the age of 16 suffer from cataract, and among these, 200,000 (15%) are severely visually impaired or blind [1,2]. While this figure is relatively low compared to the 17 million (40%) adults who are blind due to cataract [3], the burden of disability in terms of “blind-years” is huge due to the child’s life expectancy after developing the visual disability. In addition, untreated congenital cataracts can cause permanent blindness by interfering with the focus of light on the retina, necessary for formation of neural pathways necessary for vision. This presents an enormous problem in developing countries in terms of human morbidity, economic loss, and social burden.

Pediatric cataracts are both clinically and genetically heterogeneous. About one-third to one-half of all bilateral pediatric cataracts have a genetic basis [4-6]. All three forms of Mendelian inheritance have been observed, and the most frequently seen in non-consanguineous populations is autosomal dominant (AD) transmission [7,8]. To date, at least 34 loci in the human genome have been reported to be associated with various forms of pediatric cataract. Of the mapped loci, mutations have been identified in 22 specific genes including encoding crystallins (*CRYAA* [9], *CRYAB* [10], *CRYBA1* [11], *CRYBA4* [12], *CRYBB1* [13], *CRYBB2* [14], *CRYBB3* [15], *CRYGC*, *CRYGD* [16], and *CRYGS* [17]), cytoskeletal proteins (*BFSP1* [18] and *BFSP2* [19]), membrane proteins (*GJA3* [20] and *GJA8* [21], *MIP* [22] and *LIM2* [23]), transcription factors (*HSF4* [24], *PITX3* [25], and *MAF* [26]), glucosaminyl (N-acetyl) transferase 2 (*GCNT2* [27]), chromatin modifying protein-4B (*CHMP4B* [28]), and *TMEM114* [29]. On the basis of current studies, mutations in about half of affected families occur in crystallins, a quarter in connexins, and the remainder is evenly split between membrane proteins, intermediate filament proteins, and transcription factors. However, the relative contribution of these classes of genes to pediatric cataracts in India is still unclear.

Crystallins are the major cytoplasmic proteins of the lens and their stability and appropriate interactions are critical for lens transparency. Crystallin genes encode more than 95% of the water soluble structural proteins present in the vertebrate lens and their encoded proteins account for more than 30% of its mass. In 1894, Morner first separated bovine lens proteins into three soluble fractions and one insoluble fraction [30]. The soluble fractions consisted of α-, β-, and γ-crystallins, which are found in all vertebrate lenses and are referred to as “ubiquitous crystallins.” In the mature human lens, α-crystallin makes up roughly 40%, β-crystallin 35%, and γ-crystallin 25% of the total crystallin protein. The β- and γ-crystallins form a super family as they share a common two domain structure composed of four extremely stable, torqued β-pleated sheets termed “Greek key” motifs. At least 13 functional crystallin genes have been identified in humans, and of these, 10 major crystallin genes have been associated with pediatric cataract [12,31].

To clarify the relative contributions of mutations in the crystallin genes to congenital cataracts in the Indian population, a systematic screening of the 10 crystallin genes associated with cataract was performed in a large panel of congenital cataract patients.

## Methods

### Family ascertainment

Patients with a positive family history of childhood cataract were recruited from the Pediatric Ophthalmology Clinic, Aravind Eye Hospital (AEH), Madurai, India. The family members were interviewed to obtain a detailed medical, ophthalmic, and family history and were included in the study based on their availability and willingness. Patients with a history suggestive of intrauterine infection such as rubella, complicated cataract, and traumatic cataract were excluded from the study.

Ophthalmologic examination included the best corrected visual acuity (Snellen’s), slit lamp biomicroscopy, intraocular pressure measurement by applanation tonometry, and fundus examination. Corneal diameter and axial length were measured when indicated. In addition, the probands were examined by a physician to check for the presence of other systemic abnormalities. Photographs of significant findings were taken depending upon the cooperation of the patient. Normal subjects were drawn from the General Ophthalmology Clinic of the Aravind Eye Hospital (AEH) matching the ethnic distribution of the pediatric cataract patients. Ethics approval for the study was obtained from the Institutional Review Boards of the AEH and Combined Neuroscience IRB at the National Eye Institute, and the study was performed in accordance to the tenets of Declaration of Helsinki. Informed consent was obtained from the participating members in the study. DNA was extracted from venous blood as previously described [32].

### Molecular analysis

One affected representative individual from each of the identified families was chosen for mutation analysis. Primers were designed to amplify the entire coding exons and 10–30 bp of the flanking intronic sequences of the 10 crystallin genes - *CRYAA*, *CRYAB*, *CRYBA1*, *CRYBA4*, *CRYBB1*, *CRYBB2*, *CRYBB3*, *CRYGC*, *CRYGD*, and *CRYGS* (primer sequences available upon request). Single strand conformational polymorphism (SSCP) analysis was employed in the detection of mutations. The amplicons were denatured at 98 °C for 5 min and electrophoresed at 700–800 V for 8 −12 h at room temperature on 6%–12% polyacrylamide gels. Additives including glycerol (5%–10%) were used in gels. The gels were silver stained according to the modified protocol of Bassam et al. [33].The samples whose electrophoresis patterns differed from those of the controls were sequenced. The polymerase chain reaction (PCR) product was purified using Ampure and CleanSeq purification kits (Agencourt, Beckman, Beverly, MA) on a Beckman NX MC automated workstation (Beckman). Sequencing of the DNA was performed using chain terminator chemistry with a Big Dye terminator v3.1 Cycle Sequencing Kit (Applied Biosystems, Foster City, CA) on an ABI 3130 DNA analysis system.

The DNA sequence was analyzed and compared with the reference sequence using the Seqman program of the DNASTAR analysis package (Lasergene, Madison, WI). If a nonsynonymous sequence change that results in a change in the amino acid sequence of the protein was found, available family members were then analyzed for cosegregation of the genotype with the phenotype. In addition, 100 control chromosomes were analyzed to confirm association of a previously described mutation, and 200 normal control chromosomes were analyzed to confirm the association of a novel sequence variation with the disease phenotype. If available, the presence or the absence of a restriction site was employed to confirm the cosegregation of sequence variation among the family members and in the control population. When no suitable restriction site was identified, direct sequencing was performed to analyze the cosegregation of the genotype with the disease phenotype.

### Homology modeling

Modeling of the human βγ-crystallins, γC- and γS-crystallin, was performed by homology modeling based on correspondent crystal coordinates for murine protein structures (Brookhaven Protein Database [PDB] files: 2v2u and 1zw0) as the structural templates [34]. For each model, primary sequences were aligned by the method of Needleman and Wunsch [35] and incorporated in the program, Look version 3.5.2 [36,37] for three-dimensional structure prediction. Finally, two models of γC- and γS-crystallin monomers were built by the automatic segment matching method in the program, Look, followed by 500 cycles of non-bound energy minimization [38]. These models were used to predict the effect of mutations in *CRYGC* and *CRYGS*. In addition, structures of two human βγ-crystallins, γD- and βB2-crystallin, (PDB files: 1ytq and 1hk0, respectively) were chosen to model mutations identified in *CRYGD* and *CRYBB2*. The conformations of the proteins with mutations Q155X (βB2-crystallin), R168W (γC-crystallin), R140X (γD-crystallin), and S39C (γS-crystallin) were modeled and refined by self-consistent ensemble optimization (500 cycles) [37].

## Results and Discussion

A total of 60 patients below the age of 25 years with a positive family history of pediatric cataract were registered for the study. The families were coded as CCW1-CCW60 according to the order collected. The mode of inheritance was inferred from the pedigree based on the vertical or horizontal transmission as dominant or recessive inheritance, respectively. About 53% (n=32) had AD inheritance, 30% (n=18) had autosomal recessive (AR) inheritance, and in 17% (n=10), the inheritance pattern could not be determined. The age of onset was recorded as the age at which the disease was first noticed by the child’s parents or first documented by a clinician. Depending on the age of onset, the pediatric cataract was classified as congenital, infantile, or juvenile cataract. Congenital cataracts present at birth were observed in 56% (n=33) of the patient families. An additional 8% (n=5) had infantile cataracts that developed within the first three years of life, and cataracts that developed within the second decade of life and were characterized as juvenile cataract were observed in 33% (n=20). Finally, 3% of the families (n=2) had a variable age of onset. Fifteen families also revealed other associated ocular disorders such as microcornea, microphthalmia, myopia, and anterior and/or posterior lenticonus.

The mutations identified in the crystallin genes analyzed are summarized in Table 1. The analysis allowed the identification of causative nine mutations in 10 of the 60 families. Of the nine individual mutations identified, three are novel and six have been reported previously. These include six missense mutations (CRYAA-R12C, R21W, R54C, CRYAB-A171T, CRYGC-R168W, and CRYGS-S39C), two nonsense mutations (CRYBB2-Q155X, CRYGD-R140X), and one splice mutation that was identified in two separate families (CRYBA1-IVS3+1G>A). This suggests that mutations in crystallin genes might account for approximately 16.6% of pediatric cataracts in the study population.

**Table 1:** Summary of crystallin mutations identified in south Indian families.

**Gene**	**Family ID**	**Base change**	**Amino acid**	**Blosum 80**	**Age of onset**	**Cataract phenotype**	**Other ocular anomalies**	**Restriction site employed in conformation**	**Controls screened**
*CRYAA*	CCW-46	c.104 C>T	R12C	−6	Birth	Nuclear	Microcornea	ApaLI +	100
	CCW-36	c.130 C>T	R21W	−5	Birth	Nuclear	Microcornea	MspI -	100
	CCW-55	c.230 C>T	R54C	−6	Birth	Nuclear	Microcornea + Microphthalmous	HpyCH4V +	100
*CRYAB*	CCW-22	c.557G>A	A171T*	0	Birth	Lamellar	-	HpyF10VI -	100
*CRYBA1*	CCW-01	IVs3+1G>A	-	-	Birth	Lamellar, Floriform (Variable)	-	Nla III +	100
	CCW-57	IVs+1G>A	-	-	Birth	Lamellar	-		
*CRYBB2*	CCW-19	c.495C>T	Q155X	−8	5–10 years	Cortical + pulverulent	-	-	50
*CRRYGC*	CCW-33	c.502 C>T	R168W	−5	Birth	Lamellar	-	-	50
*CRYGD*	CCW-45	c.418C>T	R140X*	−8	Birth	Nuclear	-	-	100
*CRYGS*	CCW-47	c.168C>G	S39C*	−2	7–10 years	Lamellar, Sutural (Variable)	-	HpyF10VI +	100

The asterisk indicates the novel mutations identified in this study. The “+” and “−” indicate the gain and loss of a restriction site as a result of the mutation.

### Summary of mutations in α-crystallin genes and the corresponding proteins

The α-crystallin gene family consists of two similar genes coding for αA-crystallin (*CRYAA* located on chromosome 21q22.3) and αB-crystallin (*CRYAB* on chromosome 11q22.1) sharing 57% sequence identity [39]. The first exon of each gene encodes 60 amino acids consisting of a repeated 30 amino acid motif while the second and third exons code for regions homologous to the small heat shock proteins [39,40].

### Description of mutations in *CRYAA*, their inheritance and associated phenotypes

**Figure 1:**
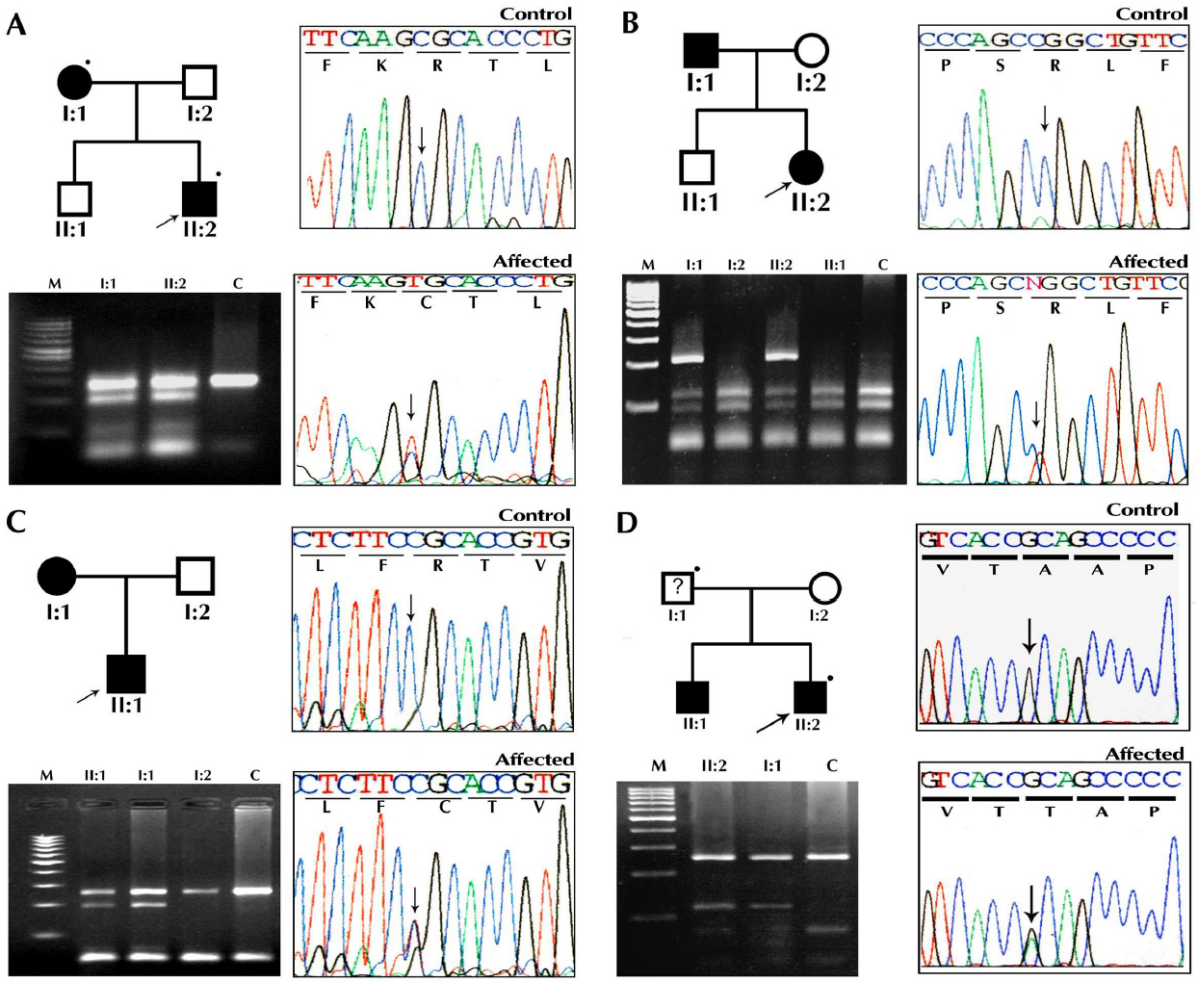
Pedigree, electropherogram, and restriction fragment length polymorphism of ADCC families with a mutation in the α-crystallin gene. In the pedigree, the square symbol represents males while the circle symbol represents females. A circle with a slash denotes a deceased individual, and a blackened symbol denotes an affected individual. The dot on the upper right corner of the symbol means a sample was available from that individual, and the arrow denotes the proband. **A**, **B**, **C** show mutation analyses of *CRYAA*. **A**: Family CCW46 shows a heterozygous c.104 C>T resulting in a novel ApaLI restriction site (mutant allele−191 and 63 bp, wild type−254 bp) **B**: Family CCW36 shows a c.130 C>T change that results in the loss of the MspI restriction site (mutant allele-254 bp, wild type-116, 90, and 48 bp) **C**: Family CCW55 shows the gain of a novel HpyCH4V site cosegregating with the affected individual heterozygous for c.230 C>T transition (mutant allele-254, 191, and 63 bp and wild type-254 bp). **D**: Family CCW22 has a mutation in *CRYAB*, c.557G>A. This mutation results in the loss of a HpyF10VI site (mutant allele-258 and 118 bp, wild type-258, 77, and 41 bp). M denotes 100 bp DNA ladder, and C denotes unrelated control.

**Figure 2:**
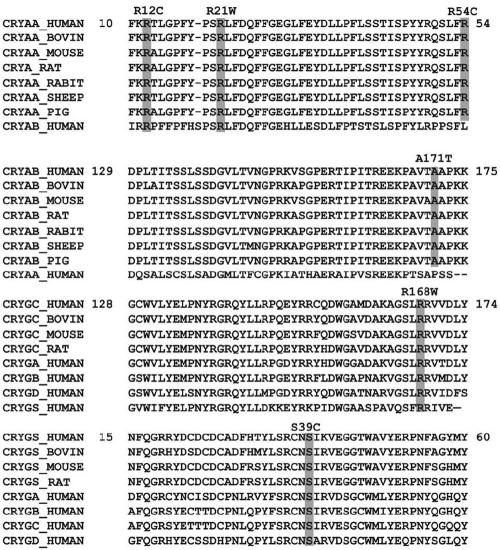
Protein sequence alignments. Protein sequence alignment showing sequence conservation of CRYAA (top alignment), CRYAB (second alignment), CRYGC (third alignment), and CRYGD (bottom alignment) among closely related species and members of the α- and γ-crystallin gene families.

In the present study, mutation analysis of *CRYAA* revealed three missense mutations, c.104 C>T (R12C) in family CCW46, c.130 C>T (R21W) in family CCW36, and c.230 C>T (R54C) in family CCW55 (Figure 1A-C). It is interesting to note that the affected members of these three families developed bilateral congenital nuclear cataract in association with microcornea except for family CCW36 (R21W) where the affected members were also diagnosed with microphthalmia. All three mutations identified in this study occur outside the small heat shock protein core domain (sHSP), and the arginines at positions 12, 21, and 54 are highly conserved among α-crystallin (Figure 2). Previous studies on the predicted topology of *CRYAA* suggest that the NH_2_-terminal region is involved in quaternary subunit interactions [41], raising the possibility that inappropriate disulfide bridge formation by the cysteine in the R12C and R54C mutations might lead to insolubility of the protein. Another possibility is that αA-crystallin has been shown to have a strong tendency to maintain a constant net charge through evolution [42]. The mutations identified in this study result in the replacement of the basic amino acid, arginine, by a neutral one, which also could affect protein–protein interactions.

An increasing number of C*RYAA* mutations have been associated with cataract formation in humans and mice, exhibiting both autosomal dominant and recessive inheritance. Interestingly, most of the mutations in αA-crystallin involve arginine residues and act in a dominant fashion (R12C [42], R21W/L [43,44], R49C [45], G98R [46], and R116C/H [9,43]). These mutations have led to isolated cataracts or cataracts associated with microcornea, microphthalmia, and/or iris coloboma [9,43,44]. The changes, R12C and R21W, have previously been reported to result in the cataract-microcornea syndrome [43]. The cataract phenotypes of these two mutations are similar, consisting of central, zonular opacification with varying involvement of the anterior and posterior pole. The identical mutations identified in this study result in nuclear cataracts with microcornea. The *CRYAA* mutation, W9X, in humans and the R54H mutation in mice have been reported to cause AR cataract [47,48]. During the preparation of this manuscript, Khan et al. [49] reported R54C to cause an autosomal recessive cataract where the family members homozygous for the mutation had congenital total cataract with microcornea and bilateral punctuate cataracts were observed in heterozygous carriers of the mutation. In contrast, the R54C mutation reported in this study is associated with autosomal dominant congenital nuclear cataract (ADCC) associated with microcornea. In summary, the *CRYAA* mutations identified in this study result in nuclear cataract in association with microcornea in all three families and associated with additional microphthalmia in one family.

### Description of mutations in *CRYAB*, their inheritance and associated phenotypes

A novel mutation, A171T, was identified in *CRYAB* in family CCW22 (Figure 1D), the proband was the only available clinically confirmed affected family member available for this study. The proband and his unexamined father revealed a heterozygous c.557G>A change. The proband showed lamellar cataract phenotype with no ocular or systemic abnormalities.

αB-crystallin is widely expressed in several non-ocular tissues including cardiac and skeletal muscles [50]. Several mutations in *CRYAB* have been described including one associated with cataract and desmin related myopathy (Arg120Gly) [51] and in patients with desmin-related myofibrillar myopathy without cataract (464delCT and G151X) [52]. In 2001, the *CRYAB* mutation, K150fs, was associated with isolated posterior polar cataract in an English family [10]. P20S and D140N have also been reported to cause isolated forms of ADCC [53], but it is not clear why some mutations in *CRYAB* cause muscle system disorders and/or cardiovascular defects and other mutation causes isolated cataract.

The A171T mutation identified in this study could not be confirmed to cosegregate with cataracts due to the lack of available family members. However, analysis of 200 normal control chromosomes showed the absence of such a change. This mutation also lies in a phylogenetically conserved region in the COOH-terminii of αB-crystallin (Figure 2), which is considered necessary for the chaperone function of the protein. Most of the mutations reported in *CRYAB* are in this region, consistent with the importance of the sequence encoded in exon 3 for αB-crystallin function. Further, there have been reports of a *CRYAB* mutation associated with lamellar cataract [53] similar to the phenotype observed in the proband of family CCW22. While not definitive, all of these suggest that the A171T mutation is responsible for the cataracts in this patient.

### Summary of mutations in β-crystallin genes and the corresponding proteins

The β-crystallins are divided into seven subgroups, three basic (βB1-, βB2-, and βB3-crystallin) located on chromosome 22q11.2 and four acidic forms (βA1/βA3-crystallin located on chromosome 17q11.2, βA2-crystallin located on chromosome 2q33, and βA4-crystallin located on chromosome 22q11.2). All the β-crystallin genes except *CRYBA2* have been associated with congenital cataracts. Screening β-crystallin genes in this population revealed a splice site mutation in *CRYBA1* and a nonsense mutation in *CRYBB2*.

### Description of mutations in *CRYBA1*, their inheritance and associated phenotypes

**Figure 3:**
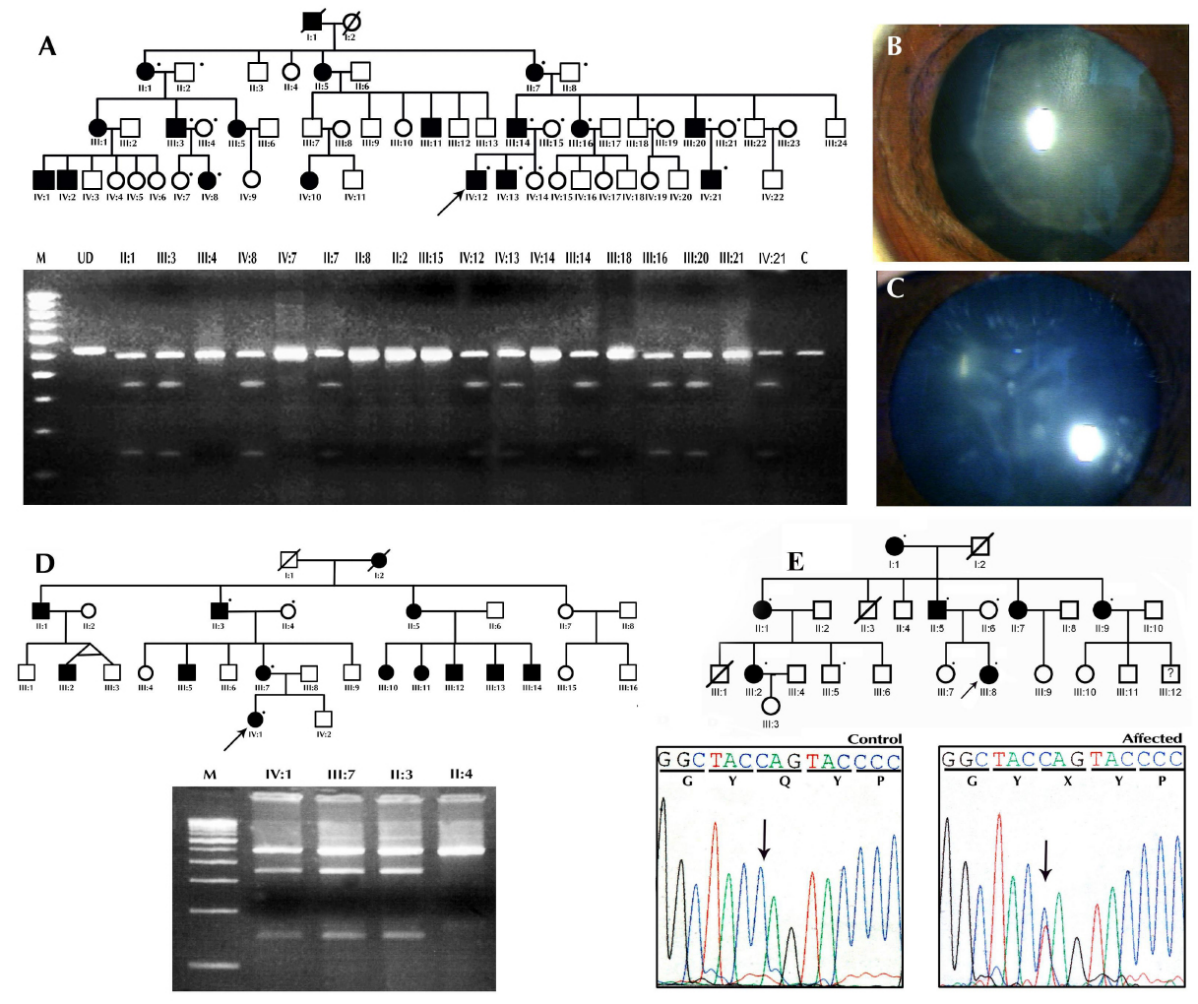
Mutation analysis of β-crystallin genes. **A**: Pedigree and RFLP analysis of family CCW1 is shown. The dot on the upper right corner of the symbol denotes a sample was available for the study. The mutation, IVS3+1 G>A, in *CRYBA1* creates a novel NlaIII restriction site. Wild type individuals display a 488 bp and 32 bp band while the affected individual display a 488 bp, 346 bp, 142 bp, and 32 bp cleavage product. The latter 32 bp DNA fragment expected to be generated from the PCR product due to the presence of a common NlaIII site cannot be visualized in the agarose gel. Lens images of individuals of family CCW-1, IV:12 aged 8 years with lamellar cataract (**B**) and individual III:20 aged 33 years (**C**), show floriform cataract respectively. **D**: Pedigree and RFLP analysis of family CCW58 with IVS3+1 G>A mutation in *CRYBA1* are shown. **E**: Mutation analysis of *CRYBB2* in pedigree CCW19 shows heterozygous C>T transition in the chromatogram that results in Q155X. M denotes 100 bp DNA ladder, C denotes unrelated control, and UD denotes undigested PCR product.

A previously reported splice site mutation (IVS3+1 G>A) was identified in two families, CCW1 and CCW57, with an AD mode of inheritance (Figure 3A,D). The disease shows complete penetrance, and the ophthalmic records confirm the opacification of the lens was bilateral in all affected members. The members whose medical records were available in these two families showed the affected individuals were diagnosed with zonular lamellar opacification. However, in family CCW1, individual IV:21 had lamellar cataract and needed to undergo surgery at two years of age while his father III:20 at 33 years old had floriform cataract and his visual acuity is 6/36 in the right eye and 4/60 in the left (Figure 3B,C).

To date, there are three mutations in *CRYBA1* that have been associated with cataracts. These include a three base pair deletion in exon 4 (∆G91) that caused AD congenital pulverulent nuclear cataract in three Chinese families, a suture sparing nuclear cataract in a Swiss family and an English family with the identical mutation for which no clinical description is available [54-57]. A splice site mutation, IVS3+1G>C, has been associated with a pulverulent cataract phenotype in a family from Brazil [58], and a G>A transition at the same position has been reported to cause lamellar, sutural cataract in an Indian family and sutural nuclear cataract with peripheral cortical opacities in an Australian pedigree [11,59]. The *CRYBA1* mutation identified in the two families (CCW1 and CCW57) in this study is a G>A transition at the 5′ donor splice site. The mutation was found to be associated with a characteristic zonular lamellar cataract. The Australian pedigree with the IVS3+1G>A mutation included an individual with a mild opacity. Similar individuals are included in our family (CCW1). The cause of intra-pedigree variability of cataract phenotype is unknown, although it could result as an effect of a modifier gene or environmental factor.

The donor splice site mutation is expected to cause disruption of βA3- and βA1-crystallins, which differ from each other only in the length of their 5′-terminal extensions. The first two exons of *CRYBA1* encode the NH_2_-terminal arm, and exons 3–6 encode the Greek key motifs [60]. As speculated by Kannabiran et al. [11], the effect of this mutation will result in skipping of the donor splice junction or recruitment of a cryptic splice site that would affect the proper formation of the Greek key motifs.

### Description of mutations in *CRYBB2*, their inheritance and associated phenotypes

**Figure 4:**
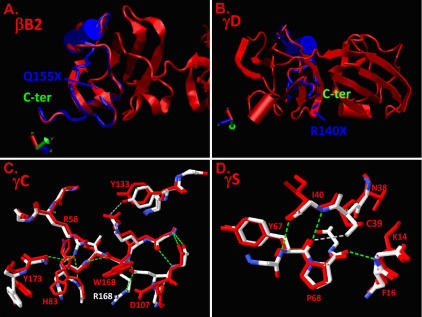
Molecular modeling of the effects of mutations presented in Table 1 on βγ-crystallin structures. Normal structures are shown in red and the overlaid mutant structures are shown in blue (truncations) and blue and white (substitutions). **A**: The normal βB2-crystallin structure is shown in red, and the Q155X mutation is predicted to remove the COOH-terminal 51 amino acids shown in the blue overlay. **B**: The normal γD-crystallin structure is shown in red and the R140X mutation is predicted to remove the COOH-terminal 34 amino acids shown in the blue overlay. **C**: The normal γC-crystallin structure in the vicinity of the R168R mutation is shown in white with positive charges shown in blue and negative charges in red. The overlaid R168W mutant is shown in red, demonstrating the substitution of the polar arginine residue by the apolar tryptophan and consequent displacement of D107. **D**: The normal γS-crystallin structure in the vicinity of the fragments of 3D structures of mutant proteins (γC-crystallin) and S39C (γS-crystallin) mutations are shown in red. Hydrogen-bonding patterns of the mutant γC- and γS-crystallin structures are shown by dashed lines (green).

As previously reported, the nonsense mutation, Q155X (c.495C>T), in exon 6 of *CRYBB2* was identified in family CCW19 (Figure 3E). Affected individuals in this three-generation family have cortical cataracts with pulverulent opacities. All affected individuals developed poor vision between the ages five and seven years. The mutation (Q155X) is predicted to remove the final 51 amino acids, effectively deleting ~90% of the fourth Greek key motif and the entire COOH-terminal arm of the protein as demonstrated in Figure 4A. The loss of these amino acids is predicted to remove the last three β-strands in the sequence, resulting in an unstable molecule.

Previously, six geographically distinct families have been reported with the same chain-termination mutation (Q155X) in *CRYBB2* [14,61-65]. Clinical descriptions of the cataract morphologies vary widely from cerulean cataract in American and Moroccan families [14,61] to a Coppock-like phenotype with pulverulent opacities in a Swiss family [62], punctate cataracts with cerulean opacification in an Indian family [63], and progressive polymorphic cataracts ranging from pulverulent to dot, strip, star-like, and sheet shapes in a Chinese family [64]. A Chilean family exhibited variable and disparate phenotypes ranging from pulverulent cortical opacities to a dense posterior star-shaped subcapsular cataract [65]. The cataract morphology identified in this study shows little similarity to the Swiss and Chilean families. This mutation clearly demonstrates the phenotypic heterogeneity of the disease.

### Summary of mutations in γ-crystallin genes and the corresponding proteins

The family of γ-crystallin genes is mainly located in a cluster of six highly related genes (*CRYGA-CRYGF*) on human chromosome 2q33–35 and the seventh *CRYG* gene (*CRYGS*) on human chromosome 3. Mutations in *CRYGC, CRYGD*, and *CRYGS* have been associated with congenital and juvenile hereditary cataracts [16,17,66]. Molecular analysis of these genes in this study revealed one previously reported mutation in *CRYGC* (R168W) and novel mutations in *CRYGD* (R140X) and *CRYGS* (S39C). Results of modeling the structural changes corresponding to these mutations are shown in Figure 4B-D.

### Description of mutations in *CRYGC*, their inheritance and associated phenotypes

**Figure 5:**
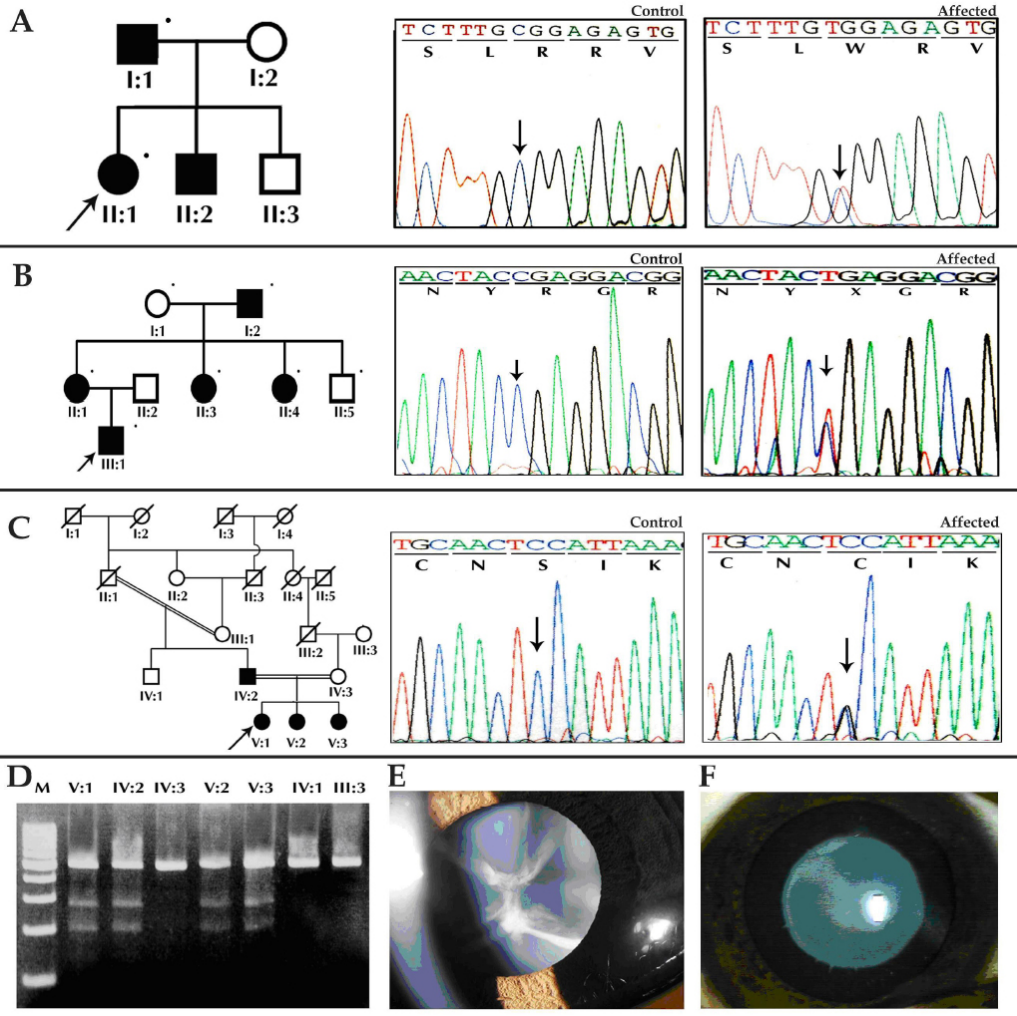
Mutation analysis of γ-crystallin genes. **A:** Family CCW-33 shows an R168W mutation in *CRYGC*. **B:** Family CCW-45 shows an R140X mutation in *CRYGD*. **C:** Family CCW47 shows a S39C mutation in *CRYGS*. **D**: RFLP analysis of *CRYGS* exon 1 shows the loss of HpyF10VI site (mutant allele-274 bp and 204 bp, wild-type allele-478 bp). **E**: Individual V:3 of family CCW47 shows sutural cataract. **F**: Individual II:1 of family CCW33 shows lamellar cataract. M denotes 100 bp DNA ladder, and C denotes unrelated control.

Two affected members of family CCW33, both having an R168W mutation, were available for study (Figure 5A). Hospital records confirmed the cataract was present at birth. Upon clinical examination, affected individual II:1 displayed a lamellar cataract phenotype (Figure 5F). R168 is highly conserved among the γ-crystallins (Figure 2). It is a hydrophilic amino acid with a positive charge and lies within the extended strand on the surface of the molecule, interacting with water. In the R168W mutant, arginine is replaced by tryptophan, a hydrophobic amino acid. Changing the solvation property of an amino acid residue on the surface of the γ-crystallin protein is predicted to diminish its solubility [67]. In addition, replacement of R168 with a tryptophan residue is predicted to result in a significant change in the conformation of residues located close to residue 168, altering the hydrogen bonding pattern as demonstrated in Figure 4C.

To date, there are three mutations reported in *CRYGC*, one insertion mutation and two missense mutations. The insertion mutation (p.C42fs) resulted in zonular, perinuclear, or polymorphic cataracts with varying degrees of opacification [68]. Of the missense mutations, T5P is reported to cause a Coppock-like cataract that presents with a dust-like opacity of the fetal nucleus [16] and R168W has been reported to cause lamellar and dense nuclear cataracts in Indian and Mexican families, respectively [69,70]. It is interesting to note that the lamellar cataract morphology observed in family CCW33 in this study is similar to that reported by Santhiya et al. [69] in that both are childhood lamellar cataracts. While both these families are from southern India, a single origin could not be confirmed.

### Description of mutations in *CRYGD*, their inheritance and associated phenotypes

A nonsense mutation in exon 3 of *CRYGD* (c.418C>T, R140X) cosegregates with cataracts in the affected members of family CCW45 (Figure 5B). Ophthalmic records of the proband confirm the opacity was present during early infancy and mainly affected the central zone or the fetal nucleus. All other affected members of the family underwent surgery at an early age. The R140X mutation is predicted to result in the loss of 34 amino acids, resulting in only partial formation of fourth Greek key motif and misfolding of the truncated protein. The truncated part of the R140X mutant is shown in blue (Figure 4B). This would presumably be followed by precipitation of the protein resulting in opacification of the lens. The two previously reported nonsense mutations in *CRYGD*, Y134X in Danish families [42] and W157X reported in an Indian family [69], also occur in exon 3, affecting the fourth Greek key motif and causing nuclear cataract. The R140X mutation identified in this study results in a similar nuclear cataract phenotype.

### Description of mutations in *CRYGS*, their inheritance and associated phenotypes

A novel missense mutation (c.168C>G, S39C) was identified in exon 2 of *CRYGS* in the two-generation family CCW47 (Figure 5C). Affected individuals in this family show a progressive juvenile onset cataract. Deterioration of vision of affected family members began at seven years of age. Initially, one eye had more severe opacification than the other. The sequence variation, c.168C>G, in *CRYGS* cosegregates with the trait in the kindred studied and is absent in 200 ethnically matched normal control chromosomes (Figure 5D). Serine is highly conserved at position 39 (Figure 2). Replacement of serine at position 39 by cysteine as shown in Figure 4D is predicted to break three hydrogen bonds including the one involving S39 directly (shown in white). Although both serine and cysteine are neutral hydrophilic amino acids, it is possible that the new cysteine residue might form inappropriate homocysteine bonds in an oxidizing environment.

This is the second report of a *CRYGS* mutation to be associated with cataract. In the previous report, a G18V mutation was identified in a Chinese family affected with progressive polymorphic cortical cataract. The opacities in this Chinese family varied not only between family members but also between eyes in the same patient [17]. Similar findings were observed in family CCW47 in which the severity of the cataracts varied from one eye to another of the same patient. Individual V:3 shows sutural opacities in the left eye while the right eye showed no opacification (Figure 5E). Individual V:1 and V:2 had lamellar opacities and underwent surgery at the end of the first decade of their lives. Phenotypic variation in the size and density of the opacities and in their position was also observed among other affected family members.

**Figure 6:**
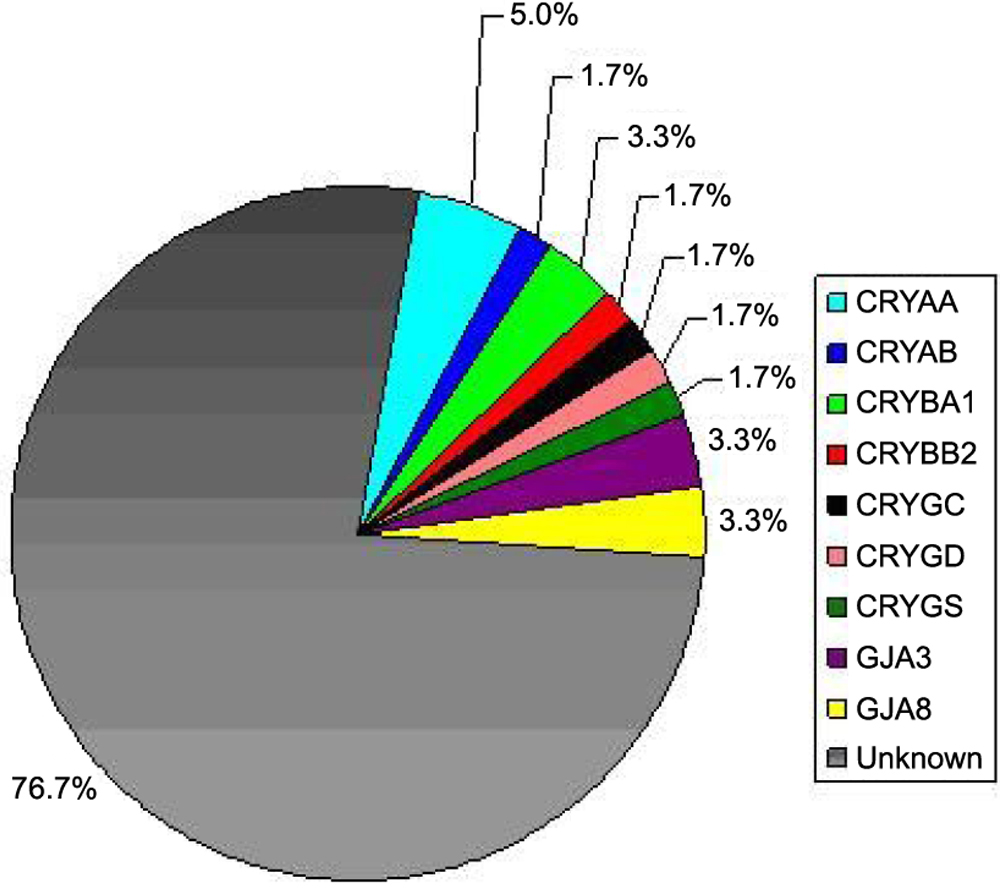
Frequency of crystallin and connexin mutations in the south Indian population. Pie chart showing the frequencies of crystallin and connexin mutations in the south Indian popultaion as seen in this study and in references [71] and [72].

In summary, this study identified mutations in 10 of 60 families from southern India affected by pediatric cataracts. *CRYAA* was the gene most commonly involved with causative mutations identified in 5% of the families while both *CRYAA* and *CRYAB* together accounted for 6.6% of pediatric cataracts in the families studied. Identical mutations were identified in *CRYBA1* (IVS3+1G>A) in two large families, suggesting that this is a common mutation site for this gene. No mutations were detected in *CRYBA4*, *CRYBB1*, or *CRYBB3*. It is notable that all of the mutations identified in this study cause autosomal dominant cataract. Previously, the same set of families has been screened for mutations in connexin genes (*GJA3* [71] and *GJA8* [72]), which revealed mutations in four families. The frequency of involvement of the 10 crystallin genes and two connexin genes analyzed in the Indian families is summarized in Figure 6. Taken together, these 12 genes account for almost one-quarter of inherited cataracts in this group of families from southern India.

In contrast, causative mutations in these 12 genes (10 crystallin and two connexin) were not identified in over 76% of the families (n=46). While it is certainly possible that causative mutations occurring in these genes were missed in some cases or would not be detected by sequencing (e.g., large deletions or mutations in promoter or intronic splice enhancer sequences), it seems unlikely that these would account for a large fraction of the families without identified mutations. Thus, the pathogenesis of the majority of cataract families in India seems not to be accounted for mutations by the coding exons of crystallins or connexins, and mutations in lens crystallins and connexins appear to be underrepresented in this population. A previous Australian study involving systematic screening for mutation in five crystallin genes (*CRYAA*, *CRYBA1*, *CRYBB2*, *CRYGC*, and *CRYGD*) in 38 pedigrees has reported only two mutations (CRYBA1-IVS3+1G>A and CRYGD-P23T). Given that studies on two independent populations describe a low frequency of crystallin mutations, perhaps the dominance of crystallins in the literature is not a reflection of the true distribution of cataract genes, but rather it represents the attractiveness of the crystallins as candidate genes for screening studies. However the possibility cannot be excluded that mutations in one of the other reported candidate genes such as membrane intrinsic proteins (*MIP* and *LIM2*), intermediate filament protein (*BFSP2*), transcription factors (*MAF*, *PITX3*, and *HSF4*), and glucosaminyl (N-acetyl) transferase 2, I-branching enzyme (*GCNT2*) may be the major cause of hereditary cataracts in India. Alternatively, it is possible that currently unidentified genes may be a more significant cause of cataracts than previously thought. Extensive linkage analysis and screening of additional candidate genes will assist in discrimination between the above hypotheses.
